# Human Autoinflammatory Diseases Mediated by NLRP3-, Pyrin-, NLRP1-, and NLRC4-Inflammasome Dysregulation Updates on Diagnosis, Treatment, and the Respective Roles of IL-1 and IL-18

**DOI:** 10.3389/fimmu.2020.01840

**Published:** 2020-08-25

**Authors:** Sara Alehashemi, Raphaela Goldbach-Mansky

**Affiliations:** Translational Autoinflammatory Diseases Section (TADS), Laboratory of Clinical Immunology and Microbiology (LCIM), National Institutes of Allergy and Infectious Diseases (NIAID), National Institutes of Health (NIH), Bethesda, MD, United States

**Keywords:** inflammasome, autoinflammatory diseases, NLRP3, pyrin, NLRC4, NLRP1, GSDMD

## Abstract

Recent research has led to novel findings in inflammasome biology and genetics that altered the diagnosis and management of patients with autoinflammatory syndromes caused by NLRP3-, Pyrin-, NLRP1-, and NLRC4-inflammasomes and spurred the development of novel treatments. The use of next-generation sequencing in clinical practice allows for rapid diagnosis and the detection of somatic mutations that cause autoinflammatory diseases. Clinical differences in patients with NLRP3, pyrin, and NLRP1 inflammasomopathies, and the constitutive elevation of unbound free serum IL-18 that predisposes to the development of macrophage activation syndrome (MAS) in patients with gain-of function mutations in *NLRC4* led to the screening and the characterization of novel diseases presenting with constitutively elevated serum IL-18 levels, and start to unravel the biology of “high IL-18 states” that translate into the use of biomarkers that improve diagnosis and monitoring of disease activity and investigations of treatments that target IL-18 and IFN-gamma which promise to improve the management and outcome of these conditions. Lastly, advances in structural modeling by cryo-electron microscopy (cryo-EM) of gasdermin, and of NLRP3- and NLRC4-inflammasome assembly, and the characterization of post-translational modifications (PTM) that regulate inflammasome activation, coupled with high-throughput screening (HTS) of libraries of inflammasome-inhibiting compounds, promise a new generation of treatments for patients with inflammasome-mediated diseases.

## Introduction

Genetically-defined autoinflammatory diseases present with systemic and organ-specific inflammation caused by Mendelian defects in critical innate immune pathways (1, 2). The discovery that gain-of-function (GOF) mutations in NOD-like receptors (NLR) that form IL-1β activating inflammasomes cause systemic autoinflammatory diseases led to the successful repurposing of targeted anti-cytokine treatments that block IL-1 signaling and spearheaded precision medicine in autoinflammation.

Recent insights into the structure and function of the four inflammasomes, NLRP3-, pyrin-, NLRC4, and NLRP1, that so far have been associated with human disease, revealed differences in assembly, and their downstream function. These discoveries shed light on pathomechanisms that may cause the phenotypic differences between the inflammasome-mediated diseases. In particular, the unique association of the NLRC4 inflammasome with extremely high serum IL-18 levels and of the NLRP1 inflammasome with keratinocyte differentiation defects point to the differential roles of the respective inflammasomes in hematopoietic vs. non-hematopoietic cells and tissues.

Although the autoinflammatory diseases spectrum continues to expand and now includes syndromes caused by type-I IFN, IL-17, TNF, and IL-6 dysregulation (1, 2), this review focuses on recent updates on inflammasome biology gained by insights into the structure, post-translational modifications and differences in IL-18 cleavage that spur the development of inflammasome-specific targeted treatments. Advances in genetic diagnoses using next generation sequencing together with the emergence of novel treatment targets, promise to benefit conditions with inflammasome-amplified inflammation beyond autoinflammatory syndromes, that include malignancies, metabolic, vascular, and neurodegenerative diseases.

## Updates on NLRP3 and NAIP/NLRC4 Inflammasome Activation and IL-1 Release

**Figure 1:**
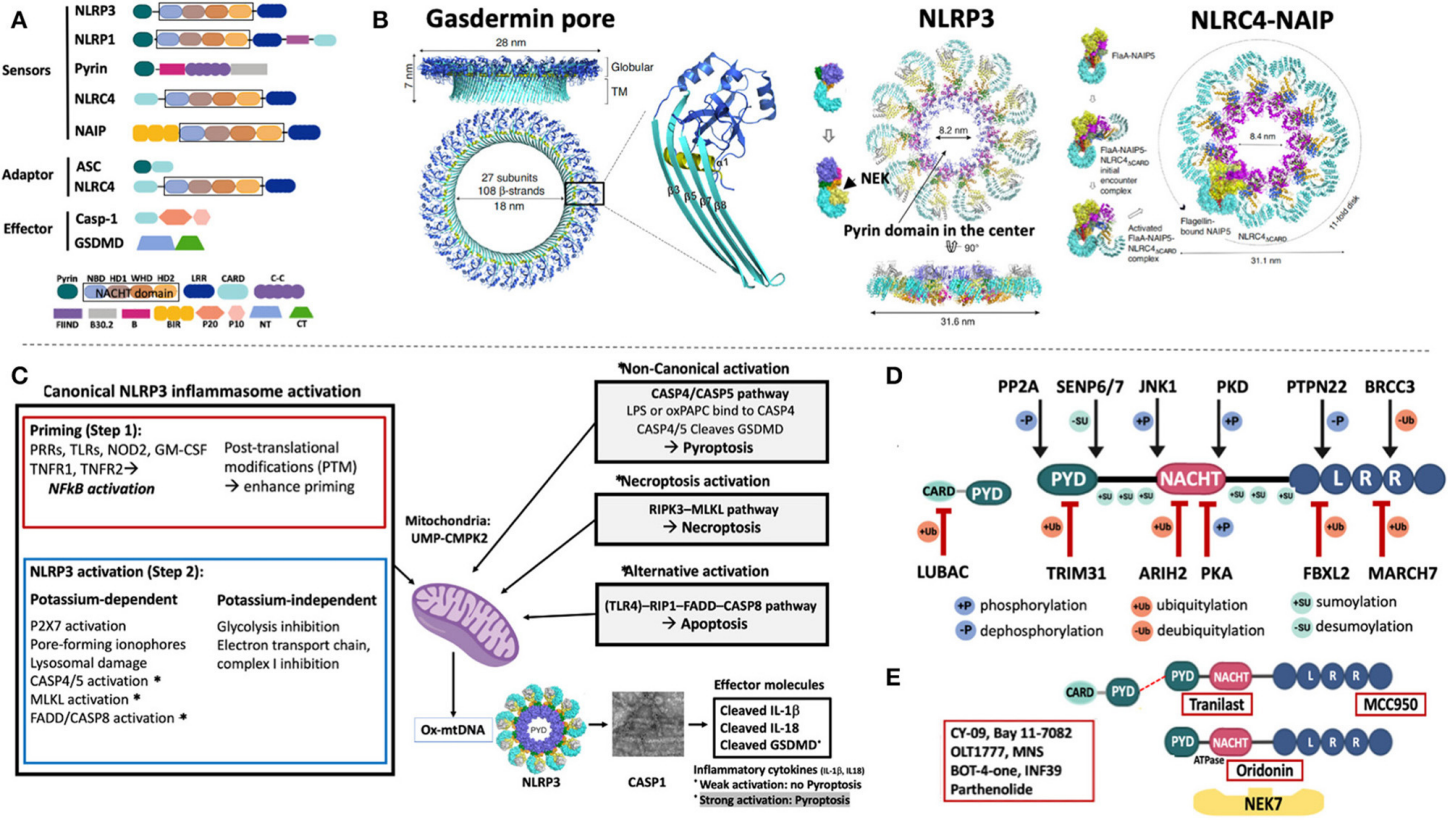
Domain structure and activation of inflammasomes that cause human diseases. **(A)** The inflammasome sensors are tripartite proteins with an amino-terminal PYRIN (PYD), CARD, or BIR domain, a nucleotide-binding NACHT domain, and a carboxy-terminal leucine-rich repeat (LRR) domain. Intracellular sensors, including NLRP3, NLRP1, Pyrin, and NLRC4/NAIP, oligomerize upon stimulation and recruit and activate pro-Caspase-1 (pro-Casp-1), which cleaves proinflammatory cytokines (pro-IL-1β, pro-IL-18, not shown), and gasdermin-D (GSDMD). All sensors except for pyrin, have a “NACHT domain” that includes an NBD, an HD1, a WHD1 and a HD2 domain (shown in black box). **(B)** The 3D cryo-EM structure of the gasdermin D pore is shown (left panel); 27 cleaved N-terminal gasdermin fragments assemble a 27-multimeric ring, the gasdermin pore. The cryo-EM structures of NLRP3 (middle panel) and NLRC4-NAIP (right panel) demonstrate assembly of 11 or 12 NLRP3 or NLRC4/NAIP monomers through self-oligomerization into a disc-like structure. NLRP3 binds to NIMA-related Kinase 7, NEK7; NLRC4 binds to NAIP, which is a sensor of microbial flagellin, and of components of the bacterial Type III injection system. **(C)** Inflammasome activation. *Canonical NLRP3 inflammasome activation* requires a first or “priming” step which encompasses pattern recognition receptor/cytokine induced transcriptional upregulation of pro-*IL1B* and genes of some NLRP3 inflammasome components. The second step that leads to NLRP3 activation can be K+ efflux-dependent or independent and eventually leads to mitochondrial stress and the production of oxidized mitochondrial DNA (Ox-mtDNA); its production is controlled by the rate-limiting enzyme UMP-CMPK2. *Non-canonical inflammasome activation* is triggered by caspase-4/5 in humans (and caspase-11 in mice) that cleave GSDMD but not the pro-inflammatory cytokines and induces pyroptosis without priming step 1. Furthermore, activation of the RIPK3-MLKL pathway mediates necroptosis and alternative activation through FADD-Caspase-8 induces apoptosis and triggers inflammatory cytokine release through NLRP3 activation. One hypothesis to reconcile how different NLRP3 activating signals activate the inflammasome is through the common generation of mitochondrial distress and the release of Ox-mtDNA. **(D)** Post translational modifications of NLRP3 and ASC control inflammasome activation and have become targets for drug development. In resting macrophages, the LRR domain of NLRP3 is ubiquitylated. Deubiquitylation by the deubiquitinating enzyme (DUB) BRCC3, and dephosphorylation by protein tyrosine phosphatase, PTPN22 promote NLRP3 oligomerization while the E3 ubiquitin ligases, MARCH7, and FBXL2, ubiquitinate the NLRP3 LRR domain to inhibit NLRP3 inflammasome activation. The NACHT domain is modified by phosphorylation and dephosphorylation at serine residues, p.S194 and p.S293 by JNK1, and PKD, respectively, which activate, while phosphorylation or ubiquitylation at sites modified by PKA and ARIH2, respectively, inactivate the NLRP3 inflammasome. Modifications of the PYD domain at a Lys48-linked ubiquitylation site by the E3 ubiquitin ligase, TRIM31, cause proteasomal degradation of NLRP3 whereas dephosphorylation at p.S5 by PP2A and desumoylation by SENP6/SENP7 promote NLRP3–ASC, NLRP3 PYD–PYD interactions and inflammasome activation. Six conserved sumoylation loci keep NLRP3 in a resting state; desumoylation by SENP6/7 promotes NLRP3 activation. **(E)** Presumed drug-NLRP3 interaction sites are depicted. The MCC950 mechanism of action is unknown, while Tranilast, a tryptophan analog binds to the NACHT domain and inhibits NACHT-NACHT interaction between NLRP3 monomers. Oridonin binds to the NACHT domain and blocks NLRP3 and NEK7 interaction. A group of direct NLRP3 inhibitors including OLT1177 (Dapansutrile), a β-sulfonyl nitrile compound, block the NACHT ATPase activity. Residue numbers refer to human protein (ENST00000336119). **(A,B)**: B, Pyrin B-box; B30.2, Pyrin B30.2 domain; BIR, Baculovirus IAP-repeats; CARD, Caspase Recruitment Domain; Casp-1, Caspase 1; C-C, coiled-coiled domain; CT, C- terminal domain of gasdermin; FIIND, Function to Find Domain; HD1, Helical Domain 1; HD2, Helical Domain 2; LRR, Leucine Rich Repeat; NACHT, NAIP/C2TA/HET-E/TP1; NBD, nucleotide-binding domain; NT, N- terminal domain of gasdermin; PYD, pyrin domain; P20, protein 20; P10, protein 10; WHD, Winged Helix Domain. **(C)**: CASP1, caspase-1; CASP4/5, caspase-4/5; CASP8, caspase-8; FADD, Fas-Associated protein with Death Domain; GM-CSF, Granulocyte-monocyte colony stimulating factor; GSDMD, Gasdermin D; LPS, Lipopolysaccharide; MLKL, mixed-lineage kinase domain-like protein; NFkB, nuclear factor-kB; NOD2, nucleotide-binding oligomerization domain-containing protein 2; oxPAPC, oxidized phospholipid 1-palmitoyl-2-arachidonoyl-sn-glycerol-3-phosphorylcholine; P2X7, purinoceptor 7; PRR, Pattern recognition receptor; RIP1, receptor-interacting protein 1; RIPK3, receptor interacting protein kinase 3; TLR, Toll-like receptor; TNFR1, tumor receptor factor receptor 1; TNFR2, tumor receptor factor receptor 2; UMP-CMPK2, Cytidine Monophosphate Kinase 2. **(D,E)**: ARIH2, Ariadne homolog 2; BRCC3, BRCA1/BRCA2-containing complex subunit 3; FBXL2, F- box/LRR- repeat protein 2; JNK1, c-Jun N-terminal kinase 1; MARCH7, membrane-associated RING finger protein 7; NEK7, NIMA related kinase 7; *PKA*, protein kinase A; PKD, protein kinase D; *PP2A*, protein phosphatase 2A; *PTPN22*, protein tyrosine phosphatase, non-receptor type 22; SENP6/SENP7, Sentrin/SUMO-specific protease 6/7; TRIM31, tripartite motif containing protein 31.

Inflammasomes are intracellular sensors that regulate host defense, cell homeostasis, and cell death. Upon activation they recruit and activate caspase-1, which cleaves the proinflammatory cytokines pro-IL-1β, pro-IL-18, and gasdermin-D (GSDMD) (Figure 1). Cryo-electron microscopy (Cryo-EM) provided stunning models by deconvoluting the structure of the gasdermin pore and the assembly of the disc-like structures that initiate the assembly of the NLRP3 and NLRC4 inflammasomes that shed light on the enigma of IL-1 (and IL-18) release from activated monocytes (3) and on the mechanism of pyroptotic cell death (4, 5).

### Cryo-EM Structure of Gasdermin-D, NLRP3 and NLRC4, and Novel Insights Into IL-1 Release

High-resolution 3D-evaluations of the molecular structure of gasdermin- D, a key regulator of pyroptosis (4) revealed that 27 cleaved N-terminal fragments of murine GSDMA3 (6) or human GSDMD (7) assemble a 27-multimeric ring that forms a pore which gets inserted into the cell membrane and serves to release cleaved IL-1β and IL-18 from a respective cell (8–11) (Figure 1B, left panel). If the inflammasome-activating signal is weak, the endosomal sorting complexes required for transport (ESCRT) system (12) can repair and close the pores (13, 14). A strong activating signal that exceeds the ESCRT repair capacity causes leakage of cell content through the pores that results in pyroptotic cell death (15, 16). In contrast to caspases-3, -6, and -7 mediated apoptosis (17, 18) which is not immunogenic, pyroptotic cells leak immunogenic contents and illustrate the importance of tight regulation of gasdermin-D cleavage through enzymatically active caspase-1.

In fact, the conventional view of regulation of caspase-1 activity was challenged in a recent study examining caspase-1 activation. In contrast to the model that caspase-1 tetramers, composed of two p20 and two p10 subunits (p20/p10), are activated through auto-processing (19), the authors demonstrate that full-length p46 and transient p33/p10 caspase complexes are enzymatically active and that auto-processing of p33/p10 generates inactive p20/p10 subunits (20). While therapies that block caspase-1 have failed, knocking out GSDMD in murine models of CAPS, and FMF prevented disease development (21, 22), suggesting GSDMD as a potential treatment target.

In late 2001/2002, the NLRP3 inflammasome was the first cytoplasmic sensor discovered to be linked to human disease (23), but only recently cryo-EM has solved the structure that provided insights into mechanisms that ignite its assembly. In the cryo-EM model, NEK7, a mitotic kinase that had been identified as critical in activating the NLRP3 inflammasome (24–26), was bound to the LRR domain. cryo-EM models suggest that it binds adjacent NLRP3 monomers and licenses self-nucleation through formation of an 11 or 12-subunit disc-like complex (endecamer or dodecamer) (27) (Figure 1B, middle panel) which assembles ASC and caspase-1 filaments that cleave IL-1b (not shown).

Cryo-EM of the (NAIP)-NLRC4 inflammasome illustrates a different activation mechanism. Microbial flagellin and components of the bacterial Type III injection system bind to NAIP, which undergoes a conformational change that enables binding of NAIP to an NLRC4 protomer and exposes a “catalytic” oligomerization interface that recruits another NLRC4. Self-oligomerization continues until 10-11 NLRC4 protomers and one NAIP form a disc that can recruit caspase-1 (3) (Figure 1B, right panel). NLRC4 can also oligomerize by helical assembly and form filaments similar to ASC and caspase-1 (28, 29). These oligomerization processes allow for rapid signal amplification during inflammasome activation. The biological roles of the disc-like vs. filament assembly are currently not understood.

### Activation of the NLRP3 Inflammasome and Characterization of Post-translational Modification (PTM) Sites That Regulate Inflammasome Activation and Provide Novel Treatment Targets

Over the last 17 years studies of the NLRP3 inflammasome activation revealed complex signaling pathways that lead to inflammasome assembly (Figure 1C). To summarize current insights, the canonical activation of the NLRP3 inflammasome requires two signals. The first signal is mediated by a range of “triggers” (i.e., DAMPS and PAMPS, PRPs, TLRs, NOD2, TNFR1) that cause NF-kB mediated upregulation of *IL1B* transcription (30, 31). The second signal leads to caspase-1 activation and is triggered by mechanisms that cause potassium-efflux including through P2X7 channel activation, exposure to pore-forming ionophores, lysosomal damage, activation of the non-canonical caspase 4/5 pathway, necroptosis by the RIPK3-MLKL pathway (32–34), and activation through the alternative RIP1-FADD-CASP8 pathway which are described in more detail in the figure legend of Figure 1C (35). Potassium-independent inflammasome activation is mediated by inhibition of the oxidative transport chain and glycolysis (36, 37) (Figure 1C) that ultimately causes release of oxidized mitochondrial DNA (ox-mtDNA) that can directly bind and activate NLRP3 (38). The enzymatic activation of the mitochondrial deoxyribonucleotide kinase, UMP-CMPK2, that can synthesize ox-mtDNA (39) in a rate-limiting step, and has recently been characterized. UMP-CMPK2 can be activated in a TLR–MyD88/TRIF–IRF1 dependent manner, which has become a target for drug development. Lastly, cell compartment disruption can activate the inflammasome by disassembly of the trans-Golgi network which serves as scaffold for NLRP3 (20). This process can be induced by mitochondria-associated membrane (MAM) localization to Golgi membranes, which causes diacylglycerol accumulation and the recruitment of protein kinase D, a phosphorylase that phosphorylates NLRP3 and triggers its release from MAMs and subsequent oligomerization (40). Mulitple other PTMs that modify NLRP3 inflammasome activation illustrate the complex regulation of the NLRP3 inflammasome and are described in Figure 1D.

To summarize, ubiquitination of PTM sites in the LRR domain in resting macrophages, inactivate the NLRP3 inflammasome, whereas deubiquitylation, and dephosphorylation of respective LRR sites activate it. Ubiquitylation, phosphorylation and sumoylation sites in the NACHT and PYD domain further modify NLRP3 function. Six conserved sumoylated loci (p.K88, p.K133, p.K204, p.K552, p.K652, p.K689) keep NLRP3 in a resting state (41). Mutations in the SUMO conjugation motifs, p.R137H, p.P651S, p.E690K, and p.E692K, cause CAPS and in p. D90Y, p.E206G, and R556X, an unspecified autoinflammatory syndrome thus suggesting that sumoylation defects may cause disease by lowering the NLRP3 activation threshold. The role of PTM in regulating NLRP3 inflammasome activation triggered the characterization of enzymes that may be subject to inhibition by small molecules and inspired high throughput screening (HTS) of chemical compound libraries for small-molecules that inhibit the activation of the NLRP3 inflammasome.

So far, three small molecule compounds have been identified that have been or are currently tested in clinical trials (Figure 1E), although their exact mechanisms of action remain elusive. 1. Tranilast is used in an ongoing single arm prospective study in CAPS (NCT03923140), its effect on pharmacokinetics and pharmacodynamics when co-administered with febuxostat in patients gout and hyperuricemia (NCT00995618) have also been evaluated (42). 2. OLT1177 (Dapansutrile) showed efficacy when topically applied as gel in a randomized, double-blind controlled study in osteoarthritis (NCT02104050). In an open-label, proof-of-concept, phase 2a trial, dapansutrile reduced target joint pain in a dose-dependent manner in adult patients with acute gout flares (EudraCT 2016-000943-14) (43). It is currently tested in a phase 1b trial in patients with heart failure (NCT03534297) and in a phase 2, open label study in Schnitzler's Syndrome (NCT03595371) (44). 3. Studies with MCC950 (also: CP-456,773) were temporarily discontinued due to off-target effects including liver toxicity in patients with rheumatoid arthritis (45–47).

Lastly, CRISPR/Cas9, a third-generation genome editing tool, that can specifically disrupt or repair disease-causing genes by a single gRNA-directed Cas9 nuclease (48) raises hopes for more definitive treatments of inflammasome mediated diseases. In addition to viral delivery systems of the CRISPR/Cas9 system to respective cells that include adeno-associated virus (AAV)-mediated delivery systems which have been tested in murine models of hypercholesterolemia and Duchenne muscular dystrophy (49–51), non-viral delivery systems using modified lipid nanoparticles have been explored. One study used an optimized cationic lipid-assisted nanoparticle (CLAN) system that can encapsulate mCas9 and guide NLRP3 (CLAN_mCas9/gNLRP3_), to disrupt *NLRP3* gene expression in bone marrow derived macrophages (BMDM) *in vitro*, and *in vivo* when administered by injections in murine models of LPS-induced septic shock, MSU-induced peritonitis and high-fat-diet-induced diabetes (52). The uptake of CLAN particles in predominantly phagocytosing macrophages and neutrophils suggest that the system, if safe, may provide a novel treatment strategy in the future.

### Recent Developments on the Genetic Diagnosis and Management of NLRP3 Inflammasome Mediated Diseases, Caps (FCAS, MWS, and NOMID/CINCA)

**Figure 2:**
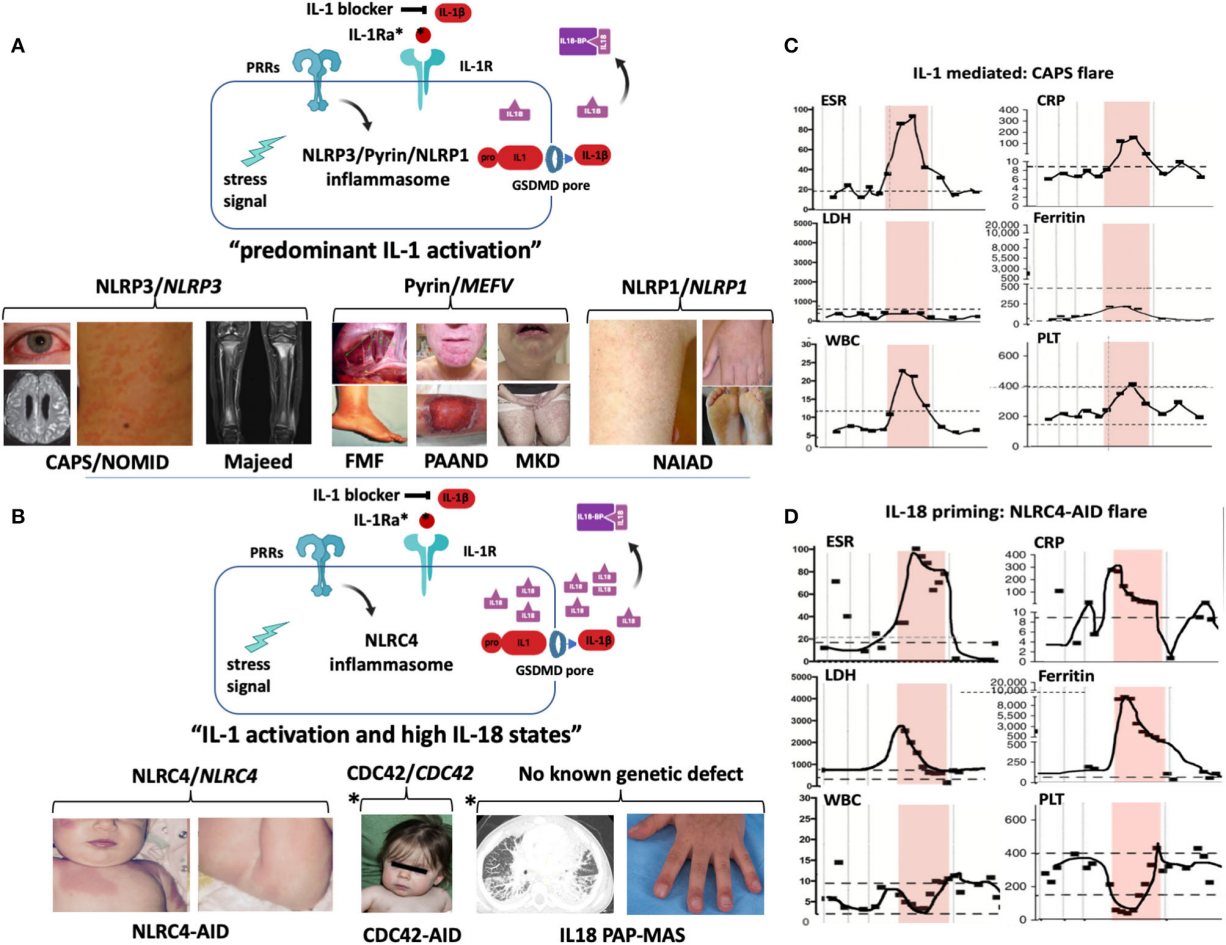
Inflammasomes and inflammasomopathies. GOF mutations in Inflammasome sensors (NLRP3, Pyrin, NLRC4, NLRP1) cause systemic autoinflammatory diseases. While GOF mutations in NLRP3, pyrin, and NLRP1 lead to predominantly IL-1b activation **(A)**, GOF mutations in NLRC4 lead to IL-1b activation and very high IL-18 levels **(B)**. **(A)** GOF mutations in *NLRP3* cause the cryopyrin-associated autoinflammatory diseases (CAPS) (images depict conjunctivitis, MRI with leptomeningeal enhancement in NOMID patient with aseptic neutrophilic meningitis and neutrophilic urticarial rash) and secondary NLRP3 inflammasome activation by recessive LOF mutations in *LPIN2* has been linked to the generation of a metabolic triggers/stress (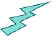) that cause Majeed syndrome (depicted is sterile osteomyelitis of the growth plates, the disease is not discussed in the text). Additive GOF mutations in pyrin cause familial Mediterranean fever (FMF) (depicted are abdominal adhesions that can develop in chronic sterile peritonitis and erysipelas-like erythema of the ankle in FMF) or Pyrin-Associated Autoinflammatory with Neutrophilic Dermatosis (PAAND) (depicted is cystic acne and pyoderma gangrenosum in a patient with neutrophilic dermatitis), and secondary pyrin inflammasome activation is caused by LOF mutations in *MVK* that cause Mevalonate kinase deficiency (MKD) (depicted is lymphadenitis and a papular rash). GOF mutations in *NLRP1* cause NLRP1-Associated Autoinflammation with Arthritis and Dyskeratosis (NAIAD) (depicted is follicular dyskeratosis, hyperkeratosis of the soles and lesions on left hand). **(B)** GOF mutations in *NLRC4* cause the NLRC4-Associated Autoinflammatory Disorders (NLRC4-AID) associated with often ultra-high serum IL-18 expression which predisposes to the development of MAS [images depict the rash from a patient with NLRC4-associated macrophage activation syndrome (MAS)]. Other high IL-18 states include *CDC42* mediated autoinflammatory disease (depicted are mild facial dysmorphisms including frontal bossing and nasal bridge depression); IL-18 PAP-MAS (a subset of SOJIA-ILD), an autoinflammatory disease without known genetic defect (depicted are a chest CT scan with interstitial lung disease and clubbing of the fingernails). For the 2 latter diseases, increased NLRC4 inflammasome activation as cause of the high IL-18 has not been demonstrated. **(A,B)** Inflammatory markers during MAS and CAPS flares differ. Flare episodes in patients with CAPS **(C)** differ from flare episodes in patients with high IL-18 levels (shown in a patient with NLRC4-AID in **D**). ESR and CRP are similarly elevated in both diseases, in MAS other features include elevated ferritin and LDH, and cytopenias (leukopenia, thrombocytopenia); while in CAPS, ferritin and LDH increase little if at all and patients develop leukocytosis and thrombocytosis. (The shaded area shows longitudinal laboratory markers collected during a 2-week period during a disease flare). CAPS, Cryopyrin-Associated Periodic Syndrome; CDC42, Cell Division Control protein 42 homolog; FMF, Familial Mediterranean Fever; IL18-BP, IL-18 binding protein; IL-1Ra, IL-1 receptor antagonist; MKD or HIDS, Mevalonate Kinase Deficiency or Hyper-IgD Syndrome; NAIAD, NLRP1 Associated Autoinflammation with Arthritis and Dyskeratosis; NLRC4-AID, NLRC4-Associated Autoinflammatory Disorders; NOMID, Neonatal-Onset Multisystem Inflammatory Disease; PAAND, Pyrin-Associated Autoinflammatory with Neutrophilic Dermatosis; PAP-MAS, Pulmonary Alveolar Proteinosis-Macrophage Activation Syndrome; PRR, Pattern recognition receptor.

Autosomal-dominant, heterozygous GOF mutations in *NLRP3* cause the disease-severity spectrum of the predominantly familial cold induced autoinflammatory syndrome (FCAS), Muckle Wells syndrome (MWS), and the mostly sporadic severe phenotype Neonatal-onset Multisystem Inflammatory Disease (NOMID) (Figure 2A). While the familial mutations are germline mutations, rare sporadic cases of FCAS and MWS and up to 50% of NOMID patients acquire somatic *NLRP3* mutations in pluripotent cells during early embryogenesis (gonosomal inheritance) that are not detected by Sanger sequencing (53). Somatic mutations in more than 5% of transcripts in blood are detected by next generation sequencing (NGS); however, deep sequencing and subcloning may be required to identify lower frequency mutations (54). Somatic *NLRP3* mutations can be acquired in bone marrow myeloid progenitor cells; in rare cases they can cause adult-onset CAPS, that presents with neutrophilic urticaria, fever, conjunctivitis, and arthralgia (55, 56).

IL-1 blockade with anakinra, canakinumab, and rilonacept is standard of care in CAPS with a well-established safety profile. Adverse events include injection site reactions (mostly anakinra) and non-serious skin, urinary tract or GI infections that do not require treatment discontinuation (57–60). A study assessing the safety and efficacy of canakinumab in 33 young children between 1-month and 5-years-of age with mostly FCAS and MWS (*n* = 29); 4 children had NOMID, reported that the treatment was well-tolerated in the young children treated (61). Disease flares post-vaccination are well-documented in MKD/HIDS (62) and together with concerns regarding vaccination efficacy and safety in other autoinflammatory disease patients receiving biologics led to recent studies in the field. The administration of canakinumab to healthy subjects 2 weeks prior to influenza and meningococcal vaccinations demonstrated that similar protective antibody titers developed in subjects receiving canakinumab and those who did not (63). Vaccination of 17 CAPS patients with polysaccharide or conjugate pneumococcal vaccines, led to disease flares in 12 patients, who all received polysaccharide vaccines (64) and a retrospective survey in 17 patients (5 CAPS, 4 MKD, 1 FMF, 7 sJIA) on IL-1 or IL-6 blockade who received live vaccines (varicella, MMR, oral polio, and yellow fever), recorded disease flares when IL-1 blocking treatment was held for vaccination and possible vaccine-induced infections (one of 5 developed varicella zoster, one of 8 post-MMR pneumonia and 1 of 1 diarrhea post-oral polio vaccination) (65). In the absence of larger studies providers need to balance the risks and benefits.

## Pyrin and NLRP1 Inflammasomes and Associated Diseases

### The Pyrin Inflammasome

Pyrin is encoded by *MEFV* and contains PYD, bZIP (transcription domain), B-Box (zinc finger), CC (coiled-coil), and B30.2/SPRY domains (Figure 1A). Pyrin requires the adaptor ASC to recruit and activate caspase-1 (66–68). PTMs modulate the pyrin inflammasome through geranylgeranylation and phosphorylation and microbes evolved to exploit these mechanisms to manipulate virulence and host defense (69). This pivotal paper laid the ground for further explorations of the role of PTMs in activating the pyrin inflammasome in FMF and MKD/HIDS, which are discussed below.

### Pyrin Inflammasome Mediated Diseases

Familial Mediterranean Fever (FMF), the most prevalent autoinflammatory disease, is characterized by recurrent episodes of fever, serositis, arthralgia, and monoarticular arthritis (70, 71). Recessive GOF mutations in *MEFV* confer additive pyrin inflammasome activation. Most patients are homozygous or compound heterozygous, but milder disease in heterozygosity occurs predominantly with more severe mutations (70). FMF-causing pathogenic *MEFV* mutations favor an active pyrin state. Missense mutations of serine p.S208 or p.S242 in *MEFV* cause a different phenotype that presents with recurrent episodes of neutrophilic dermatosis, fever, elevated acute-phase reactants, arthralgia, myalgia/myositis, an autosomal dominant disease that was termed pyrin-associated auto-inflammation with neutrophilic dermatosis (PAAND) (72). PAAND and studies that explored the disease pathogenesis of mevalonate kinase deficiency (MKD) or hyper-IgD syndrome (HIDS) uncovered the intriguing PTMs that regulate pyrin activation and linked MKD/HIDS to pyrin dysfunction.

Mevalonate kinase deficiency (MKD) or HIDS is a periodic fever syndrome that is caused by LOF mutations in *MVK*, the gene that encodes mevalonate kinase (MVK), which is an enzyme in the cholesterol pathway. MVK deficiency causes shortage of geranylgeranyl-pyrophosphate, which is not only an intermediate in the cholesterol pathway but is also essential for the biosynthesis of terpenes and terpenoids that are components of anti-microbials, hormones, and molecules that regulate cell differentiation and growth (73). Geranylgeranyl-pyrophosphate is a precursor to geranylated proteins, that include the GTPases, Kras, and RhoA. PTM through geranylation tethers them to the plasma membrane where they regulate TLR-mediated PI3K-Akt1 activation that downregulates MEFV expression (71). Geranylgeranylated RhoA activates phosphokinases, PKN1 and PKN2, that phosphorylate p.S208 or p.S242 on pyrin that enables the binding of a 14-3-3 protein, a member of a family of conserved regulatory proteins, that when bound to pyrin, keeps it inactive (72, 74). Thus, the absence or severe reduction of geranylgeranyl phosphate seen in patients with MKD results in lack of RhoA geranylation which keeps it “inactive/paralyzed” and prevents the protective pyrin phosphorylation that would allow the protective binding of 14-3-3 (74). The pathomechanism of MKD illustrates the link between “metabolic disturbances/stress” and the pyrin inflammasome activation and provides a plausible explanation for the phenotypic similarities between FMF and MKD (60) (Figure 2B).

### The NLRP1 Inflammasome and Autoinflammatory Diseases

The NLRP1 inflammasome was last to be associated with human disease. In contrast to NLRP3 and pyrin, NLRP1 has a FIIND domain, a PYD and a CARD domain and can activate caspase-1 independent of the adapter ASC (Figure 1A). A Cryo-EM model is currently not available, but in contrast to the other disease-causing inflammasomes, NLRP1-inflammasome activation is uniquely dependent on proteasomal degradation. Pathogen-derived proteolytic toxins (75, 76) including lethal factor secreted by *Bacillus anthracis* (75) or IpaH7.8, an E3 ubiquitin ligase secreted by *Shigella flexneri* (76), or host endogenous proteinases (77, 78) modify NLRP1. This process exposes a ubiquitylation site, that initiates proteaseome-dependent degradation of NLRP1. Recent insights have been summarized in “functional degradation model” of NLRP1 that suggests that cleavage of the FIIND domain releases an N-terminal, NBD–LRR–FIIND(ZU5) fragment and a small C-terminal fragment that includes the C-terminal component of the FIIND domain and a CARD domain, or FIIND(UPA)-CARD peptide. Multiple liberated C-terminal fragments self-assemble into an oligomer that serves as a platform for inflammasome assembly and caspase-1 maturation (75). The authors suggest that NLRP1 is a sensor for proteases and pathogen effectors, that can directly induce proteasomal degradation of NLRP1 (76).

Mutations in the PYD domain in *NLRP1* cause the pre-cancerous conditions, palmoplantar carcinoma, familial keratosis lichenoides chronica, and inherited corneal intraepithelial dyskeratosis without systemic inflammation (79–81) and point to a role of NLRP1 in keratinocyte differentiation (82). In contrast, GOF mutations between the NACHT and the LRR domain and at the autolytic cleavage domain, FIIND, cause NLRP1-associated auto-inflammation with systemic inflammation, arthritis, and dyskeratosis. The systemic features respond to IL-1 blockade; the skin manifestations respond to retinoic acid and vitamin A (83). These conditions suggest activation differences of the NLRP1 inflammasome in keratinocytes and monocytes (Figure 2C).

## The NLRC4 Inflammasome and the Biology and Treatment of “High IL-18 States”

### The NLRC4 Inflammasome

NLRC4, a tripartite protein with a CARD, a NACHT and an LRR domain (Figure 1A) binds to the NLR, NAIP. Mice have five Naips, humans have one NAIP, thus challenging the translation of murine findings and may explain a lack of data on human endogenous triggers of the NLRC4 inflammasome. Interestingly, actin polymerization defects seem to play a role in NLRC4 activation (84). NLRC4 can assemble as filamentous (85) or as disc-like structures (86).

### IL-18 Biology and the Association of High IL-18 States With the Development of Macrophage Activation Syndrome (MAS)

The discovery of *de novo* GOF mutations in *NLRC4* presenting with systemic inflammation, high serum IL-18 levels and recurrent macrophage activation syndrome (MAS) linked the NLRC4 inflammasome to IL-18 activation and MAS (87, 88) and triggered exploration of the role of IL-18 in regulating monocyte and macrophage function. *In vivo*, IL-18 is bound to IL-18 binding protein (IL-18 BP) and only extremely high levels of total IL-18 result in measurable free, unbound serum IL-18 (88). Murine studies overexpressing IL-18 (88) or knocking-out of *Il18bp* (87) liked IL-18 to the development of MAS following TLR9 activation. Chimeric bone marrow transplant in mice transgenic for the human disease-causing GOF *NLRC4* mutation, p.T337S, suggest that tissues (including the gut epithelium) and not hematopoietic cells are the major source of the high serum IL-18 levels (88). Phenotypic overlap and similar cytokine profiles link MAS and primary hemophagocytic lymphohistiocytosis (HLH) caused by genetic defects in cytotoxicity (89, 90) and infection-induced MAS (91). Chimeric antigen receptor (CAR) T-cell therapy can cause a “cytokine storm syndrome” that can progress to HLH/MAS-like disease. Recent studies identified recipient macrophages as a source for IL-6, and IL-1 and nitric oxide (NO) (92) that are thought to fuel the “cytokine activation syndrome.” Patients are managed with corticosteroids and IL-6 receptor blockade (93), but treatment with IL-1 blocking agents was superior to IL-6 blockade in reducing neurotoxicity in a CAR T cell leukemia mouse models (94) suggesting a role of inflammasome activation. Murine models of free IL-18 (87, 88), HLH (95), and mice transgenic for human IL-6 (IL-6TG mice) that are challenged with LPS all develop MAS/HLH-like disease and respond to neutralization of IFNγ, which reversed HLH/MAS (91). Together these data suggest that increased IFNγ production which is downstream of IL-18, and other “cytokine storm syndromes,” may present a common end-pathway that can lead to hemophagocytosis, cytopenias and hypercoagulability, and to the progressive organ failure and high mortality seen in HLH/MAS-like disease.

### Novel Diseases Associated With High Serum IL-18 Levels

Disease-causing GOF mutations in *NLRC4* cause a clinical disease spectrum. Although systematic geno-phenotype correlations are lacking, somatic mutations in the NBD domain, p.T177A cause a NOMID-like phenotype (96); germline mutations in the WHD1 domain, p.H443P (97), and p.S445P (98), cause an FCAS-like phenotype, that may be IL-1 mediated; whereas a germline GOF mutation in the NBD domain, p.S171F, can cause MAS and thrombotic vasculopathy (99). Mutations in the HD1 domain, p.T337S (100), p.V341A (101), or in the LRR domain, p.W665C (102), can all cause MAS and early-onset enterocolitis (NLRC4-MAS), which are IL-1 and IL-18 mediated (103) (Figure 2D).

The predisposition to MAS is not seen in patients with *NLRP3* mutations, furthermore, laboratory flare characteristics differ in CAPS and NLRC4-MAS. While all patients present with ESR and CRP elevation, different from CAPS flare, in patients with NLRC4-MAS and high IL-18, lactate dehydrogenase (LDH), ferritin, and more variably transaminase (AST and ALT) levels can rise astronomically and are accompanied by cytopenia (granulo-and thrombocytopenia) and splenomegaly (Figures 2C,D). Between flares, NLRC4-MAS patients normalize ferritin, but IL-18 levels stay elevated (100). In various hyperferritinemic diseases associated with HLH/MAS that include genetically complex conditions such as systemic juvenile idiopathic arthritis (sJIA), adult-onset Still's disease, infection-induced MAS/HLH (88) or familial hemophagocytic lymphohistiocytosis (fHLH) (104), serum IL-18 levels can be constitutively elevated and/or rise only with flares.

Several newly described autoinflammatory diseases present with constitutively elevated IL-18 levels that predispose to MAS in the context of infections. GOF mutations in the C-terminal region of cell division control protein 42 homolog (*CDC42)*, p.C188Y, p.R186C, and c.576A>C p.^*^192C^*^24 (105– 107) cause an autoinflammatory syndrome with predisposition to MAS (Figure 2D). The mutations are hypothesized to affect the diarginine motif, p.R186, and p.R187, which binds to liposomes containing phosphatidylinositol 4,5-bisphosphonate (PIP2) (108) that is critical in mediating actin assembly. As actin polymerization activates the NLRC4 inflammasome in a salmonella infection model (84), a role of the NLRC4 inflammasome in CDC42 mediated AID is possible but requires further studies. In another report, a large homozygous LOF mutation in *IL18BP* causing IL-18 BP deficiency led to fulminant viral hepatitis in an 11-year-old girl. Tissue biopsy identified liver macrophages and hepatocytes as IL-18 source. Although clinical features were consistent with MAS triggered by acute hepatitis A, ferritin, free IL-18 and IFNγ levels were not reported (109). Lastly, rare patients with MAS, constitutively elevated IL-18 and interstitial lung disease and/or pulmonary alveolar proteinosis (Figure 2D), who do not have monogenic disease, present with IL-18 and IFNγ driven cytokine elevation (i.e., CXCL9 and CXCL10) in blood and in bronchoalveolar lavage, suggesting that organ-specific IL-18 dysregulation (i.e., in the liver or pulmonary system) may explain the phenotypic variability in some high IL-18 states (110–113).

### Novel Therapies for Patients With High Serum IL-18 Levels

The prominent roles of IL-18 and IFNg in the immune dysregulation of MAS is being validated in treatment studies. Recombinant human IL-18BP, tadekinig alfa, showed efficacy in a patient with NLRC4 MAS (103) and had a good safety profile in an open label phase II trial of adult-onset Still's disease. Patients were able to rapidly taper systemic steroids and achieve clinical remission (114). As suggested by murine models of HLH, the humanized anti-IFNγ monoclonal antibody, emapalumab, was efficacious and is approved in the US for treatment of primary intractable HLH (115, 116). Clinical trials evaluating safety and efficacy of emapalumab and anti-human IL-18 monoclonal antibodies in sJIA-MAS are ongoing (117, 118).

## The Role of Inflammasome Activation in Diseases Other Than Systemic Autoinflammatory Diseases

Blocking NLRP3 inflammasome improves metabolic diseases in mouse models of insulin-resistance (119), non-alcoholic steatohepatitis (120), and atherosclerosis (121), and is linked to halting progression of neurodegeneration (122), and cellular senescence (123, 124). The role of inflammasomes in modulating innate immune functions in the tumor microenvironments are well-established (125, 126) and are emerging in CAR T-cell biology (94). Blocking IL-1β reduced lung cancer incidence and mortality (127) and prevented disease progression in smoldering myeloma (128, 129). High IL-18 levels were detected in patients with gastrointestinal disease (130), breast cancers (131), and in multiple myeloma (132); NLRC4 overexpression or high IL-18 levels contributed to a poor prognosis in gliomas (133) and acute myeloid leukemia (134). Despite these data, the efficacy of inflammasome modulators as adjuvant therapy in the comprehensive treatment of human malignancies, metabolic and degenerative diseases is yet to be established in clinical settings.

## Conclusion

Inflammasomes are critical in defense against pathogens and in sensing of endogenous DAMPs signals. Tissue-specific expression and differences in their activating triggers are likely responsible for some phenotypic differences in various inflammasome-mediated autoinflammatory diseases. The role of free IL-18 levels in triggering MAS, combined with improvements in genetic testing and a growing number of targeted anti-cytokine therapies have revolutionized the diagnosis and management of autoinflammatory diseases in recent years and spearhead precision medicine in diagnosis and treatment in inflammatory and a wider spectrum of non-inflammatory diseases.

## Author Contributions

RG-M developed the outline, wrote, revised the manuscript, and the figures. SA conducted a systematic literature review, wrote, and edited the manuscript. All authors contributed to the article and approved the submitted version.

## Conflict of Interest

RG-M has received grant support from SOBI, Regeneron and Novartis. The remaining author declares that the research was conducted in the absence of any commercial or financial relationships that could be construed as a potential conflict of interest. The reviewer HH declared a past co-authorship with one of the authors RG-M to the handling editor.
